# Antiobesity potential of Piperonal: promising modulation of body composition, lipid profiles and obesogenic marker expression in HFD-induced obese rats

**DOI:** 10.1186/s12986-017-0228-9

**Published:** 2017-11-16

**Authors:** Balaji Meriga, Brahmanaidu Parim, Venkata Rao Chunduri, Ramavat Ravindar Naik, Harishankar Nemani, Pothani Suresh, Saravanan Ganapathy, Satthi Babu VVU

**Affiliations:** 1 Animal Physiology and Biochemistry Laboratory, Department of Biochemistry, Sri Venkateswara University, Tirupati, Andhra Pradesh -517502 India; 2 Present Address: Department of Bio-Technology, VSU College of Sciences, Vikrama Simhapuri University, Nellore, Andhra Pradesh -524320 India; 3 Department of Chemistry, Sri Venkateswara University, Tirupati, Andhra Pradesh -517502 India; 4 National Center for Laboratory Animal Sciences, National Institute of Nutrition (Indian Council of Medical Research), Hyderabad, India; 5 Department of Biochemistry, Center for Biological Sciences, K. S. Rangasamy College of Arts Science, Tiruchengode, Tamil Nadu India

**Keywords:** Obesity, PPARγ, Insulin resistance, *Piper nigrum*, Piperonal

## Abstract

**Background:**

Black pepper or *Piper nigrum* is a well-known spice, rich in a variety of bioactive compounds, and widely used in many cuisines across the world. In the Indian traditional systems of medicine, it is used to treat gastric and respiratory ailments. The purpose of this investigation is to study the antihyperlipidemic and antiobesity effects of piperonal in high-fat diet (HFD)-induced obese rats.

**Methods:**

Piperonal, an active constituent of *Piper nigrum* seeds, was isolated and confirmed by HPLC, ^1^H and ^13^C NMR spectroscopy. Male SD rats were fed on HFD for 22 weeks; Piperonal was supplemented from the 16th week as mentioned in the experimental design. Changes in body weight and body composition were measured by TOBEC, bone mineral composition and density were measured by DXA, and adipose tissue distribution was measured by 7 T–MRI. Plasma levels of glucose, insulin, insulin resistance and lipid profiles of plasma, liver and kidney, adipocyte hormones and liver antioxidants were evaluated using standard kit methods. Expression levels of adipogenic and lipogenic genes, such as PPAR-γ, FAS, Fab-4, UCP-2, SREBP-1c, ACC, HMG-COA and TNF-α were measured by RT-PCR. Histopathological examination of adipose and liver tissues was also carried out in experimental rats.

**Results:**

HFD substantially induced body weight, fat%, adipocyte size, circulatory and tissue lipid profiles. It elevated the plasma levels of insulin, insulin resistance and leptin but decreased the levels of adiponectin, BMC and BMD. Increased expression of PPAR-γ, FAS, Fab-4, UCP-2, SREBP-1c, ACC, and TNF-α was noticed in HFD-fed rats. However, supplementation of piperonal (20, 30 and 40 mg/kg b.wt) for 42 days considerably and dose-dependently attenuated the HFD-induced alterations, with the maximum therapeutic activity being noticed at 40 mg/kg b.wt.

**Conclusions:**

Piperonal significantly attenuated HFD-induced body weight and biochemical changes through modulation of key lipid metabolizing and obesogenic genes. Our findings demonstrate the efficacy of piperonal as a potent antiobesity agent, provide scientific evidence for its traditional use and suggest the possible mechanism of action.

## Background

The incidence of overweight and obesity is mounting worldwide in recent years and since they are predisposed to hypertension, type 2 diabetes, hyperlipidemia, nonalcoholic fatty liver and cardiovascular diseases, they gravely threaten public health [1–4]. The World Health Organization report-2014 mentions that 1.9 billion adults are overweight in the world, of which 600 million are obese. The obesity epidemic is no longer limited to western cultures, but is becoming rampant worldwide with such countries as People’s Republic of China, India, Brazil and Mexico being most widely affected [5]. Unless effective measures are taken, the prevalence of obesity of 10–15% among adults will double over the next two decades; childhood obesity is even more alarming [6, 7]. These distressing facts draw attention and necessitate intensive efforts to lower the prevalence of overweight-obesity and the burden of its clinical and monetary repercussions.

Although proper dietary pattern, exercise habits, behavioral modification and pharmacotherapy are widely recommended to prevent obesity and lifestyle-related diseases, their prevalence seems to be only increasing [8]. Many attempts have been made to correct the metabolic disparity of obesity condition, including a number of drugs like fibrates, sibutramine and Orlistat, but they have significant side effects like nausea, headache, vomiting etc. Hence, alternative therapies derived from natural products have gained attention. Certain dietary components/ herbal formulations have been in use to suppress obesity and associated ailments but many of them have not undergone adequate systematic, scientific and clinical studies [9–12].

Adipogenesis and fatty-acid metabolism are organized by PPAR and SREBP family members through transcriptional regulation of target genes [13–15]. PPAR-γ is majorly expressed in adipose tissue, PPAR-α is expressed in liver and PPAR-δ is ubiquitously expressed [16, 17]. Moreover, it is extensively implicit that the regulation of lipid metabolism and energy homeostasis is achieved through HMG-CoA reductase (HMGR), acetyl-CoA carboxylase (ACC), fatty-acid synthase (FAS), fatty-acid-binding protein-4 (Fab-4), TNF-α (Tumor necrosis factor), thermoregulatory proteins and uncoupling proteins [18]. TNF-α, one of the key mediators of the inflammatory response in obesity, is articulated by a number of mechanisms, namely, infiltrating macrophages, adipocytes in the hypertrophic adipose tissue, microglia and also neurons in the hypothalamus. One or more of the above-mentioned enzymes/proteins of lipid metabolism can be considered as potential targets to develop novel therapeutics to treat obesity. In the present study, we aimed to isolate and evaluate the antiobesity activities of piperonal, an active constituent of *Piper nigrum* seeds on high-fat diet-induced obesity.

*Piper nigrum* belongs to the tropical plant family Piperaceae, which is a rich source of diverse biologically active phytochemicals [19] and food-grade spices. Piperine, one of the major pungent constituents of *Piper nigrum*, has been found to possess interesting pharmacological effects. Increasing evidence from in vitro and in vivo studies has shown that it possesses anticancer [20, 21] and neuroendocrine modulator effects [22]. A few studies have demonstrated its expectorant, antiflatulent and cholesterol-lowering properties [23, 24]. However, its antiobesity activity remains largely unexplored. Furthermore, its protective effect on obesity-induced inflammation is unclear [25]. Therefore, the present study was aimed to isolate piperonal and evaluate its antiobesity activities in HFD-induced obese rats.

## Methods

### Chemicals

For analyzing plasma glucose and insulin, commercially available kits were procured from Stanbio Laboratory, USA and Bio-Merieux, RCS, Lyon, France, respectively. Plasma was used for analyzing total cholesterol, triglycerides, phospholipids, free fatty acids, VLDL and HDL by colorimetric methods using kits (Nicholas Piramal India Ltd., Mumbai). Orlistat (Cat No 04139) was obtained from Sigma-Aldrich. All other reagents used in the experiments were of analytical grade and of high purity. Trireagent and cDNA preparation kits were obtained from Qiagen.

### Animals and treatments

Male SD rats weighing 180–200 g were randomly divided into six groups of six each. Normal control rats were fed with normal pellet diet as per AIN-93 guidelines, while the other groups of animals were fed with HFD for 22 weeks. The composition of HFD is as previously described by us [26]. Piperonal was supplemented to selected HFD groups for 6 weeks from 16th week, as mentioned in the experimental design below. Experiments were performed at the National Center for Laboratory Animal Sciences, National Institute of Nutrition, Hyderabad, India (Regd. No. 154/RO/C/1999a/CPCSEA). Animals were placed in cages at 22 ± 2 °C, with 14–16 air changes per hour with a relative humidity of 50–60% and 12 h light/dark cycle. All procedures involving laboratory animals were in accordance with the Institute Animal Ethical Committee (IAEC No: 36 a/2012(i)/a/−CPCSEA/−IAEC/SVU/MB; Dt.01.07.2012).

### Isolation and characterization of piperonal

Piperonal was isolated at the Natural Products Division, Indian Institute of Chemical Technology, Hyderabad, India, from ethylacetate extract of black pepper seeds and purity was checked by HPLC equipped with Phenomenex Luna C18 column (150–4.6 mm, 5 m i.d.). Acetonitrile-water system was selected as mobile phase, PDA detector was used to measure the wavelength and peak position and structure was confirmed by ^1^H NMR and ^13^C NMR. The procedures used are in line with Rao et al. [27].

### Drug dosage

Piperonal weighing 1.6, 1.8 and 2.4 g, each mixed with 4 kg of HFD, was prepared to get a dose of 20, 30 and 40 mg/kg b.wt. Twenty grams of diet/day/rat were given to the groups as shown in the experimental design. The exact dose of piperonal consumed was calculated from daily food intake.

### Experimental design

1.    Normal diet control
2.    High-fat diet control (HFD control)
3. :   HFD + Orlistat (5 mg/kg b.wt)
4.    HFD + piperonal (20 mg/kg b.wt)
5.    HFD + piperonal (30 mg/kg b.wt)
6.    HFD + piperonal (40 mg/kg b.wt)

### Body weight and feed efficiency ratio

The body weight of the experimental rats was assessed once a week, while the food consumed and leftover was measured daily. For the measurement of nutrient metabolites like food intake, water intake, urine volume and fecal weight, both control and experimental rats were placed in the metabolic cages for 72 h (Techniplast, Italy).

### Body composition by Total body electrical conductivity (TOBEC)

At the end of the experiment, body composition which includes lean mass, fat-free mass, fat%, total fat (g), total body Na, K levels and water content was measured in all experimental groups by Total Body Electrical Conductivity (TOBEC) using small-animal body composition analysis system (EM-SCAN, Model SA-3000 Multi detector, Springfield, USA) as described by us previously [26]. The following body composition parameters were obtained mathematically, where E stands for total electrical conductivity. i) Total body fat: total body weight – lean body mass; ii) Fat percentage: (total body fat × 100)/total body weight; iii) Lean mass: 0.5 × E + 0.3 × total body weight; iv) Fat-free mass: 16.28 + 0.4 x E; v) Total body sodium (TB Na) (mg): (2.5 × E) + 49.1; vi) Total body potassium (TB K) (mg): (4.95 × E) + 164.4; vii) Total body water (TBW): 31.5 + 1.8 × E.

The above prediction equations have been reported to be very accurate for Wistar, Sprague Dawley, F-344 N, CFY, WKY and Holtzman rats [28].

### Estimation of BMC and BMD by DXA

The body composition of the experimental animals was assessed at the end of the experiment by Dual-X ray absorptiometry (DXA) using body composition analysis system (Halogen 1000 series). DXA data were used to compare the levels of body adiposity, bone mineral concentration (BMC) and bone mineral density (BMD) between the control and experimental groups, and calculations were made according to manufacturer’s protocols [29].

### Plasma lipid profile

At the end of the experiment, blood was collected from overnight-fasted rats by retro-orbital puncture method. Plasma was separated by centrifugation at 2500 rpm for 15 min and stored at 80 °C for further biochemical analysis. Total cholesterol (TC), HDL, VLDL, LDL and triglyceride levels (TGs) were estimated by CHOD-PAP method, GPO-PAP method; phospholipids (PLs) and free fatty acids (FFAs) were assessed as described by us previously [12].

### Blood glucose, insulin and insulin resistance

Plasma glucose was estimated using kits (Cat No 1060–500, Stanbio Laboratory, USA). Plasma level of insulin was determined using kits from Bio-Merieux, RCS, Lyon, France. Insulin resistance was calculated using the homeostasis model assessment.

### Oral glucose tolerance test (OGTT)

Oral glucose tolerance test was performed at the end of the experiment; after overnight fasting, glucose was administered orogastrically at a dose of 2 g/kg body weight and blood samples were collected from supraorbital sinus at intervals of 0, 30, 60, 90 and 120 min and glucose level was estimated [26].

### Estimation of leptin, adiponectin, lipase and α-amylase

Plasma leptin and adiponectin levels were measured by using enzyme-linked immunosorbent assay kits (Crystal Chem, Downers Grove, IL, USA), performed in duplicate, as per the manufacturer’s guidelines and expressed in ng mL^−1^. Alpha-amylase and lipase activities were determined by kinetic method using the commercial kits of Labtest, Minas Gerais, Brazil and Bioclin, Minas Gerais, Brazil, respectively [26].

### Estimation of tissue lipids

Tissue (Liver, kidney) lipids were extracted from the experimental animals as per Floch et al. [30] using a chloroform–methanol mixture (2:1, *v*/v). The tissues were rinsed with ice-cold physiological saline, dried, homogenized in cold chloroform-methanol (2:1, v/v) and the contents were extracted after 24 h. The extraction was repeated four times. The combined filtrate was washed with 0.7% KCl and the aqueous layer was discarded. The organic layer was made up to a known volume with chloroform and used for tissue lipid analysis.

### Adiposity index

Adiposity index (AI), a measure of the total weight of the visceral fat depots (epididymal, retroperitoneal and mesenteric) in the body, was determined according to Taylor and Phillips method [31] using the formula: AI = (sum of the weights of the visceral fat depots/body weight) × 100.

### Organ weights

At the end of the experiment, rats were fasted overnight and euthanized by CO_2_ inhalation and subjected to necropsy. Major vital organs like heart, lungs, liver, kidneys, mesenteric fat pads, epididymal and retroperitoneal fat pads were collected. After detailed necropsy examination, these organs were weighed and organ-to-body weight ratio was measured.

### Liver antioxidants analysis

After the completion of the experimental period, rats were anesthetized, sacrificed; the liver was excised, rinsed in ice-cold normal saline followed by ice-cold 10% KCl solution, blotted, dried and weighed. A 10% *w*/*v* homogenate was prepared in ice-cold KCl solution and centrifuged at 2000 rpm for 10 min at 4 °C. The supernatant thus obtained was used for the estimation of thiobarbituric acid substances (TBARS) [32], assay of catalase [33] (CAT), reduced glutathione (GSH) [34], superoxide dismutase (SOD) [35] and glutathione peroxidase (GpX) [36].

### RT- PCR analysis

Total RNA was isolated from adipose tissue by using tri-reagent (Sigma-Aldrich, USA) according to manufacturer’s protocol and reverse-transcribed to obtain cDNA using DNA synthesis kit (Applied Biosystems, Foster City, USA).

Twenty ng of cDNA was used for semi-quantitative PCR analysis with specific primers such as PPAR-γ, TNF-α, SREBP-1c, HMG-CoA reductase, acetyl-CoA carboxylase (ACC), fatty-acid synthase (FAS), fatty-acid-binding protein-4 (Fab-4), and uncoupling protein-2 (UPC-2). The sequences of primers are mentioned in Table 1. PCR was performed for 38 cycles using the following cycling conditions: 30 s of denaturation at 94 °C, 30 s of annealing at 59 °C and 1 min of extension at 72 °C.

**Table 1:** Primer sequences of genes used in RT-PCR

Gene	Gene Bank No	Sense	Antisense
PPAR-γ	NM_013124	CTGACCCAATGGTTGCTGATTAC	GGACGCAGGCTCTACTTTGATC
TNF-α	NM_012675	GTCGTAGCAAACCACCAAG	AGAGAACCTGGGAGTAGATAAG
FAS	NM_017332	GAGGACTTGGGTGCCGATTAC	GCTGTGGATGATGTTGATGATAGAC
Fab-4	NM_053365	TCACCCCAGATGACAGGAAA	CATGACACATTCCACCACCA
SREBP-1c	NM_012767	CGCTACCGTTCCTCTATCA	TCGCAGGGTCAGGTTCT
UCP-2	NM_019354	TAAAGCAGTTCTACACCAAGGG	CGAAGGCAGAAGTGAAGTGG
HMG-CoA R	NM_013134	GGGACCAACCTTCTACCTCAG	GACAACTCACCAGCCATCAC
ACC	NM_022193	CCTTCTTCTACTGGCGACTGAG	TAAGCCTTCACTGTGCCTTCC
β- actin	NM_031144	GGCACCACACTTTCTACAAT	AGGTCTCAAACATGATCTGG

### Histopathology of adipose and liver tissues

Liver and adipose tissues were collected from experimental rats, cut into pieces and kept in 10% formalin solution. A small piece of the tissue was sectioned with microtome, fixed on slides, stained using haematoxylin and eosin (H&E) staining procedures and observed under an optical microscope with 40X magnification and photographed.

### MRI analysis

Body fat distribution was determined by 7 Tesla MRI. The rats were placed in the magnet and fixed in a nonmagnetic device. For the detection of pure fat images, respiration-triggered water- suppressed Spin Echo sequences (repetition time: 744 ms, echo time: 12.24 ms, four averages, field view: 70 mm, matrix: 192–192, water suppression: Gauss pulse 3.9 ms, 701 HZ bandwidth) were used to obtain 30 slices (slice thickness: 2 mm) in the abdominal region. For the data analysis, 20 slices at a reproducible position covering the area from the nose to anus were considered. The area of retraperitoneal fat was visually identified in the selected slice package. Tissue volumes were calculated by multiplying the corresponding number of segmented pixels by in-plane pixel dimensions and the slice thickness.

### Statistical analysis

Results are expressed as the Mean ± S.D. All the grouped data were statistically evaluated with SPSS\19.0 software. Hypothesis testing methods included one-way analysis of variance (ANOVA) followed by Least Significant Difference (LSD) test. Significance level at *p <* 0.05, 0.01, and 0.001 was considered for various parameters to indicate statistical significance.

## Results

### HPLC and NMR analysis of piperonal

**Fig. 1:**
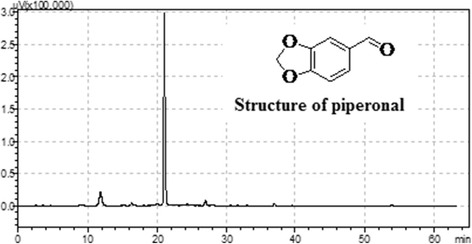
HPLC analysis of Piperonal. Pipernoal retention time 21.2 min

**Fig. 2:**
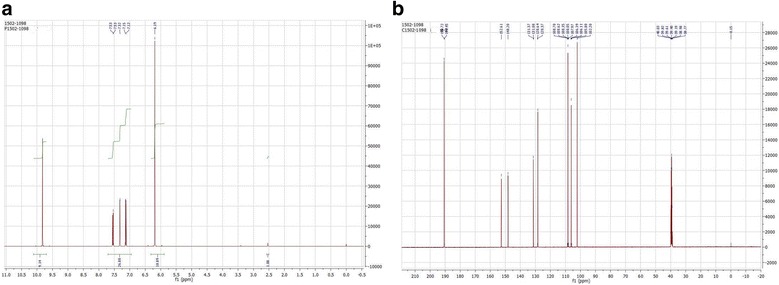
NMR analysis of isolated piperonal; (**a**) ^1^H NMR analysis; (**b**) ^13^C NMR analysis

Piperonal (benzo [d] [1, 3] dioxole carbaldehyde), also known as heliotropin is a naturally occurring bioactive compound present in *Piper nigrum,* a widely used important spice across the world. It was noted that piperonal structure is similar to piperine with a methylenedioxy ring and with molecular formula C_8_H_6_O_3_. The structure of obtained piperonal was compared with pure compound and confirmed, based on its retention time (21.2 min) by using HPLC (Fig. 1) and library search. The details of NMR studies for piperonal by ^1^H NMR are (400 MHz, CDCl_3_) δ ppm 9.82 (5, 1H, −CHO), 7.53 (dd, 1H, Ar-H), 7.32 (S, 1H, Ar-H), 7.15 (dd, 1H, Ar-H), 6.19 (S, 2H, −O-CH_2_ –O) and by ^13^C NMR (400 MHz, CDCl_3_) are 190.72, 152.6, 148.2, 131.3, 128.6, 108,7, 105.9, 102 (Fig. 2a and b) [27].

### Estimation of body composition, BMC and BMD

The changes in body weight, its composition, total body water, sodium and potassium in control and test rats are given in Table 2. There was a substantial increase in body weight, fat%, total body water, sodium and potassium level, and decrease in bone mineral concentration (BMC) and bone mineral density (BMD) in HFD-fed experimental rats fed for 22 weeks when compared to their normal control group. However, rats supplemented with piperonal (20, 30 and 40 mg/kg b.wt) for 6 weeks showed significant reduction in body weight, total fat, fat%, total body water, sodium and potassium levels and showed increased lean body mass, BMC and BMD in a dose-dependent manner when compared with HFD control group of rats.

**Table 2:** Effect of piperonal on Body weight parameters, BMC and BMD in normal and experimental obese rats

Groups	Body weight (g)	Lean body mass (g)	Total fat (g)	Fat free mass (g)	Fat %	TB H_2_O (mg)	TB Na (mg)	TB P (mg)	BMC (g)	BMD (g/sq./cm)
ND control	301.5±11.0	285.3±8.8	16.3±2.7	130.4±3.5	5.3±0.7	616.5±16.7	1023.7±28.0	2094.1±55.0	8.2 ± 0.69	0.16 ± 0.001
HFD control	506.0±9.3^a*^	391.3±9.8 ^a*^	114.5±7.1^a*^	172.8±3.9 ^a*^	22.6±1.3 ^a*^	750.5±24.1 ^a*^	1247.2±40.1 ^a*^	2536.5±79.4 ^a*^	5.5 ± 0.29 ^a*^	0.11 ± 0.002 ^a*^
HFD + Orlistat 5 mg/kg b.wt	450.1±22.8^b*^	385.3±23.8 ^b*^	64.88±7.4^b*^	170.3±9.5 ^b*^	14.3±1.7 ^b*^	782.5±52.3 ^b*^	1300.3±87.2 ^b*^	2650.3±166.8 ^b*^	7.1 ± 0.32 ^b*^	0.12 ± 0.001
HFD + Piperonal 20 mg/kg b.wt	490.3±18.0^NS^	396.8±9.4	93.5±10.2	175.0±3.7	18.9±1.4	781.1±14.9	1298.1±24.8	2636.6±49.4	6.2 ± 0.02	0.13 ± 0.003
HFD + Piperonal 30 mg/kg b.wt	456.0±15.7 ^b*^	376.9±12.6	79.0±6.6	167.1±5.1	17.2±1.2	752.3±25.7	1250.1±43.1	2542.1±84.5	6.6 ± 0.03	0.17 ± 0.002
HFD + Piperonal 40 mg/kg b.wt	454.6±12.3 ^b*^	391.8±8.5 ^b*^	62.8±8.1^b*^	173.0±3.3 ^b*^	13.7±1.5 ^b*^	798.1±18.7 ^b*^	1326.3±31.1 ^b*^	2693.0±62.0 ^b*^	7.7 ± 0.01 ^b*^	0.14 ± 0.001 ^b*^

Values are mean ± SD, *n* = 6

Values are statistically significant at *p < 0.05

^a^Significantly different from control

^b^Significantly different from HFD control

### Estimation of blood glucose, insulin and insulin resistance

Plasma levels of glucose, insulin and insulin resistance in experimental rats are represented in Table 3. HFD-fed rats showed a raise of 66, 170 and 75% in plasma glucose, insulin and insulin resistance, respectively, over their corresponding normal control rats, which was considerably reduced with piperonal supplementation in a dose-dependent manner.

**Table 3:** Effect of Piperonal on plasma glucose, insulin and insulin resistance in normal and experimental obese rats

Groups	Glucose (mg dl^−1^)	Insulin (μU ml^−1^)	Insulin resistance
ND control	90.9±7.1	6.0±0.2	3.6±.0.4
HFD Control	150.1±9.7^a*^	27.2±0.4 a*	6.3±0.2^a*^
HFD + Orlistat 5 mg/kg b.wt	103.1±4.9 ^b*^	15.8±1.4 ^b*^	4.5±0.6 ^b*^
HFD + Piperonal 20 mg/kg b.wt	140.7±7.2	24.3.7±0.44	6.0±0.2
HFD + Piperonal 30 mg/kg b.wt	131.7±2.3 ^b*^	19.7.4±0.5 ^b*^	5.1±0.2 ^b*^
HFD + Piperonal 40 mg/kg b.wt	101.5±5.5 ^b*^	17.9±0.24 ^b*^	4.8±0.3 ^b*^

Values are mean ± SD, n = 6

Values are statistically significant at ^*^ *p* < 0.05

^a^Significantly different from normal control

^b^Significantly different from HFD control

### Oral glucose tolerance test (OGTT)

**Fig. 3:**
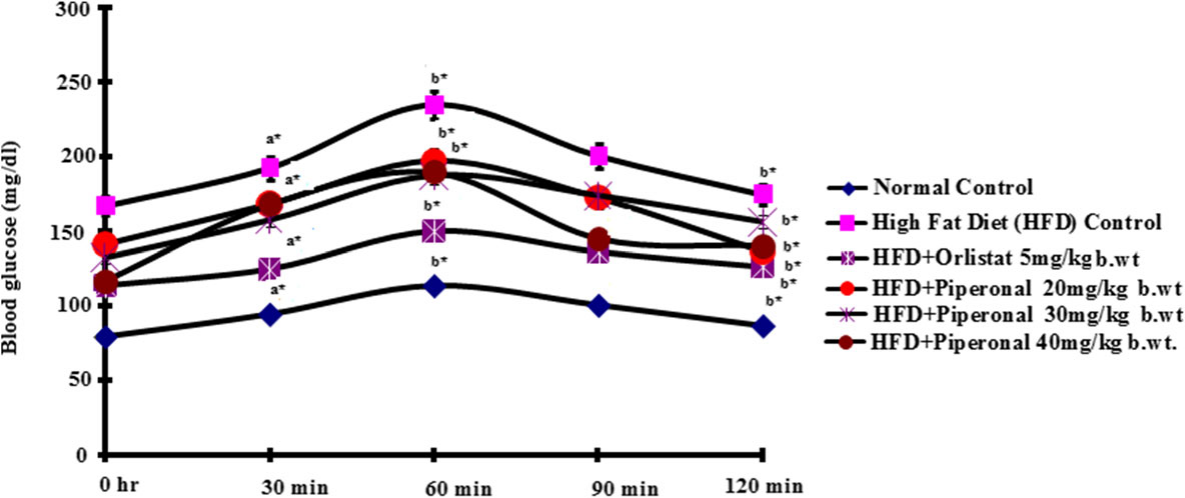
Effect of Piperonal on glucose tolerance in normal control and experimental obese rats. Values are mean ± SD, n = 6; Values are statistically significant at ^*^ *p <* 0.05; ^a*^ Significantly different from normal control; ^b*^ Significantly different from HFD control

Figure 3 presents the results of oral glucose tolerance test of the control and treated rats. When glucose was administered orogastrically, the blood glucose level in the control rats was elevated to a maximum value at 60 min after a glucose load and decreased close to basal levels at 120 min. But, in HFD-induced obese rats, peak elevated level of blood glucose was observed even after 60 min and remained high over the next 60 min. Piperonal (40 mg/kg b.wt) supplementation considerably decreased the blood glucose level after 60 min when compared with HFD-fed control rats as shown in Fig. 3.

### Estimation of leptin, adiponectin and activities of lipase and amylase

**Fig. 4:**
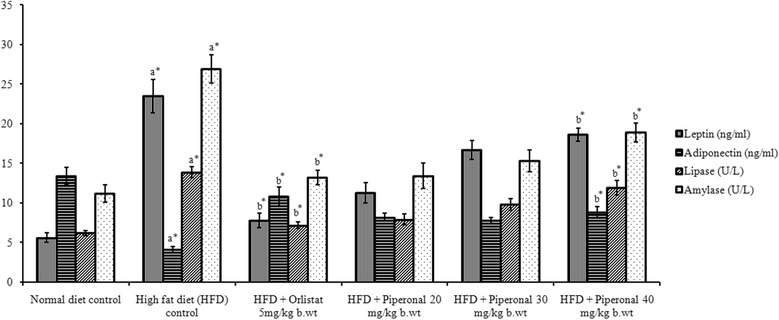
Effect of Piperonal on leptin, adiponectin, amylase and pancreatic lipase in normal control and experimental obese rats. Values are mean ± SD, *n* = 6; Values are statistically significant at ^*^ *p <* 0.05; ^a*^ Significantly different from normal control; ^b*^ Significantly different from HFD control

Figure 4 depicts the plasma leptin and adiponectin levels in control and HFD-fed rats. There was a marked (*p* < 0.05) elevation in leptin level but a decrease in adiponectin level in HFD-fed control rats as compared to normal control rats. Supplementation of piperonal (40 mg/kg b.wt) could reduce plasma leptin by 45.8% and increase adiponectin by 93.7% when compared to HFD-fed control rats. The activities of amylase and pancreatic lipase of normal and HFD-fed experimental rats are represented in Fig. 4. Supplementation of piperonal (40 mg/kg b.wt) to HFD-fed rats has brought down the activity of amylase (55%) and pancreatic lipase (59%) which was otherwise found to be much elevated in HFD-fed control rats.

### Estimation of plasma lipid profiles

When plasma lipid profiles were analyzed, HFD caused substantial alterations in TC, FFAs, TGs, PLs, HDL, LDL and VLDL of HDF-fed rats when compared to normal control rats as depicted in Table 4. Treatment with piperonal significantly (*p <* 0.05) and dose-dependently reduced the concentrations of TC, TGs, FFAs, PLs, LDL and VLDL by 42, 44, 34, 41, 59 and 43%, respectively, but increased the concentration of HDL by 76% when compared to HFD-fed control rats.

**Table 4:** Effect of Piperonal on Plasma lipid profiles in normal and experimental obese rats

Groups	TC (mg/dl)	TG (mg/dl)	HDL (mg/dl)	LDL (mg/dl)	VLDL (mg/dl)	Phospholipids (mg/dl)	Free fatty acids (mg/dl)
ND control	113.5±11.4	130.6±6.6	50.0±3.1	50.8±4.6	35.7±2.2	85.7±5.3	64.6±5.1
HFD control	311.1±20.2 ^a*^	259.5±8.5 ^a*^	26.6±2.9 ^a*^	130.8±5.9 ^a*^	81.7±4.1 ^a*^	147.9±5.7 ^a*^	116.1±4.6 ^a*^
HFD + Orlistat5mg/kg b.wt	142.6±9.8 ^b*^	136.6±11.1 ^b*^	46.8±3.4 ^b*^	55.8±5.5 ^b*^	41.6±4.4 ^b*^	100.3±5.2 ^b*^	71.2±4.3 ^b*^
HFD+ Piperonal 20 mg/kg b.wt	254.0±9.8	214.0±9.4	31.3±2.4	118.8±9.6	84.7±3.7	135.3±4.3	106.4±5.2
HFD+ Piperonal 30 mg/kg b.wt	211.0±8.2	188.5±10.4	38.7±2.1	94.7±5.8	74.1±4.6	113.2±10.6	88.6±7.6
HFD+ Piperonal 40 mg/kg b.wt	158.3±9.8 ^b*^	145.8±10.1 ^b*^	46.4±5.1 ^b*^	53.1±5.3 ^b*^	46.7±4.7 ^b*^	97.9±5.3 ^b*^	68.1±5.1 ^b*^

Values are statistically significant at ^*^ *p* < 0.05

^a^Significantly different from normal control

^b^Significantly different from HFD control

### Estimation of tissue lipids

Table 5 depicts the concentrations of TC, FFAs, TGs and PLs in liver and kidneys of control and HFD-induced obese rats. Elevated levels of TC, TGs, FFAs and PLs were noticed in liver and kidney tissues of obese rats when compared to normal control rats. Piperonal supplementation has significantly (*p <* 0.05) reduced these alterations in lipid profiles in a dose-dependent manner. On piperonal treatment, the concentrations of TC, TGs, FFAs and PLs in liver were reduced by 34, 35.6, 47.7 and 40.3%, respectively, and kidney witnessed a reduction of 35.6, 28.9, 43.6%and 41.3%, respectively, when compared with their HFD-fed control rats.

**Table 5:** Effect of Piperonal on Tissue lipid profiles (Liver, Kidney) in normal and experimental obese rats

Groups	TC (mg/g tissue)		TG (mg/g tissue)		Phospholipids (mg/g tissue)		Free fatty acids (mg/g tissue)	
	Liver	Kidney	Liver	Kidney	Liver	Kidney	Liver	Kidney
ND control	71.8±5.1	51.9±6.4	105.7±10.5	79.7±7.5	125.2±8.6	71.5±7.2	84.3±5.1	49.4±6.8
HFD control	143.8±16.4 ^a*^	132.1±7.9 ^a*^	218.3±11.1 ^a*^	145.7±8.1^a*^	203.4±12.7^a*^	150.9±9.6^a*^	180.9±6.7^a*^	119.3±9.4^a*^
HFD + Orlistat5mg/kg b.wt	76.8±6.5^b*^	73.5±5.1^b*^	119.5±13.1^b*^	87.6±7.4^b*^	134.8±8.7^b*^	80.2±6.6^b*^	87.8±5.1^b*^	61.2±3.5^b*^
HFD + Piperonal 20 mg/kg b.wt	129.8±10.6	129.6±6.2	200.1±15.3	136.4±4.5	200.3±15.1	137.9±9.3	148.9±7.1	105.1±5.4
HFD + Piperonal 30 mg/kg b.wt	118.2±16.9	109.6±6.2	182.6±7.4	123.9±7.3	175.1±8.8	123.7±4.3	135.5±11.1	84.9±5.1
HFD + Piperonal 40 mg/kg b.wt	94.6±10.1^b*^	85.6±5.6^b*^	142.1±8.1^b*^	103.1±3.9^b*^	121.1±10.4^b*^	88.1±8.3^b*^	94.8±4.1^b*^	67.9±5.8^b*^

Values are statistically significant at ^*^ *p* < 0.05

^a^Significantly different from normal control

^b^Significantly different from HFD control

### Organ weights and adiposity index

**Fig. 5:**
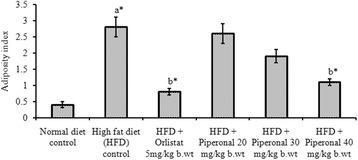
Effect of Piperonal on fat pad weights in normal control and experimental obese rats. Values mean ± S.D., n = 6. Values are statistically significant at *p < 0.05; ^a^* Significantly different from normal control; ^b^* Significantly different from HFD control

Table 6 and Fig. 5 represent organs (Liver, Heart, kidney and testis), fat pad weights (Epididymal, Retroperitoneal and Mesenteric) and adiposity index of experimental rats. Feeding on HFD considerably increased organ and fat pad weights, and the adiposity index. However, piperonal (40 mg/kg b.wt) supplementation for 42 days decreased the weights of liver, heart, kidney and testis by 28, 43.7, 30.7 and 54.1%, respectively. Similarly, fat pad weight and adiposity index showed a reduction in piperonal-treated groups.

**Table 6:** Effect of Piperonal on Organ weights in normal and experimental obese rats

Groups	Liver (g)	Heart (g)	Kidney (g)	Testis(g)	Retroperitoneal (g/100 g b.wt fat)	Mesentric (g/100 g b.wt fat)	Epididymal (g/100 g b.wt fat)
ND control	8.3±0.4	0.9±0.2	2.1±0.2	2.3±0.1	0.8±0.1	0.7±0.1	1.0±0.2
HFD control	14.0±0.2^a*^	1.6±0.2^a*^	3.9±0.11^a*^	1.8±0.1^a*^	3.5±0.4 ^a*^	2.1±0.2^a*^	2.8±0.5 ^a*^
HFD + Orlistat 5 mg/kg b.wt	10.4±0.6^b*^	1.0±0.1^b*^	2.4±0.3^b*^	2 ±0.1	1.1±0.1^b*^	1.2±0.2^b*^	1.2±0.2^b*^
HFD + Piperonal 20 mg/kg b.wt	13.3±0.5	1.3±0.1	3.7±0.3	1.8±0.3	2.9±0.1	1.9±0.2	2.3±0.3
HFD + Piperonal 30 mg/kg b.wt	11.6±0.4^b*^	1.0±0.2	3.5±0.1	1.9±0.2	2.4±0.2	1.5±0.1	1.9±0.2
HFD + Piperonal 40 mg/kg b.wt	10.0±0.6 ^b*^	0.9±0.1^b*^	2.7±0.2^b*^	2.1±0.01	1.3±0.2^b*^	0.9±0.1^b*^	1.1±0.1^b*^

Values are statistically significant at ^*^ *p* < 0.05

^a^Significantly different from normal control

^b^Significantly different from HFD control

### Liver antioxidants

**Fig. 6:**
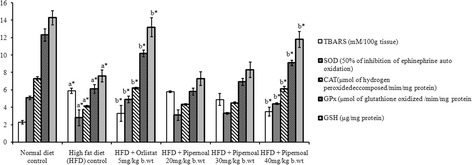
Effect of Piperonal on liver antioxidants in normal and experimental obese rats. Values mean ± S.D., *n* = 6. Values are statistically significant at *p < 0.05; ^a^* Significantly different from normal control; ^b^* Significantly different from HFD control

Figure 6 explains antioxidant levels in control and test rats. There was a two-fold increase in TBRAS level and twofold decrease in SOD, CAT, GSH and GPx activities in HFD-fed rats when compared to control rats. Supplementation of piperonal considerably and dose-dependently attenuated the above alterations to near normal levels.

### Effect of Piperonal on expression of adipogenic and lipid marker genes

**Fig. 7:**
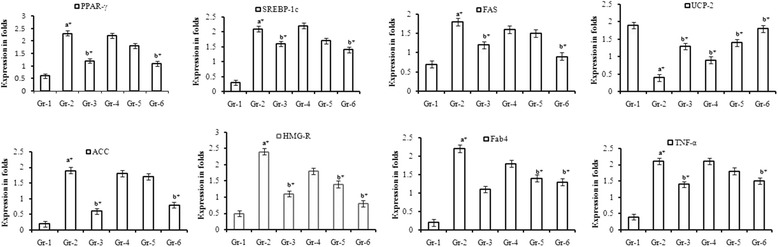
Effect of Piperonal treatment on tissue mRNA levels of adipogenic and lipogenic genes (PPARγ, TNFα, FAS, Fab-4, SREBP-1c, ACC, HMG-CoA R and UCP-2): L1- Normal control group; L2 - HFD control group; L3- HFD + Orlistat 5 mg/kg b.wt; L4- HFD + Piperonal 20 mg/kg b.wt; L5 - HFD+ Piperonal 30 mg/kg b.wt; L6 - HFD + Piperonal 40 mg/kg b.wt. Gr-1: Normal Control; Gr-2: High Fat Diet (HFD) Control; Gr-3: HFD+Orlistat 5mg/kg b.wt; Gr-5: HFD+Piperonal 30mg/kg b.wt; Gr-6: HFD+Piperonal 40mg/kg b.wt. Values are mean ± SD, n=6; Values are statistically significant at *p > 0.05; ^a^*Significantly different from Normal control ^b^*Significantly different from HFD control

To observe the diet-induced alterations at the molecular level and to evaluate the protective effects of different concentrations of piperonal, we performed semi-quantitative RT-PCR to check the expression levels of important genes associated with obesity and lipid metabolism. Compared with normal control group, HFD-fed rats showed enhanced expression of PPARγ, TNFα, FAS, Fab-4, SREBP-1c, ACC, HMG-CoA reductase and decreased expression of UCP-2. piperonal supplementation to HFD-fed rats mitigated these changes substantially in a dose-dependent manner as shown in Fig. 7.

**Fig. 8:**
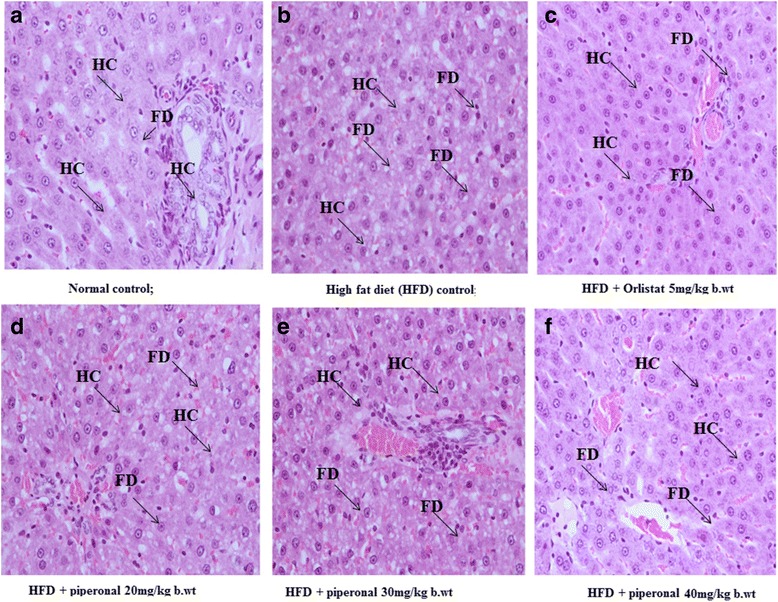
Effect of piperonal on liver histology of normal control and experimental obese rats. **a** Normal control group showing normal histopathology of the liver; (**b**) HFD control group shows increased interstitial space and distorted intercalated disc; (**c**) HFD + Orlistat 5 mg/kg b.wt; (**d**) HFD + Piperonal 20 mg/kg b.wt, (**e**) HFD + Piperonal 30 mg/kg b.wt, and (**f**) HFD + Piperonal 40 mg/kg b.wt administration showed decreased interstitial space and less distorted intercalated disc. HC: Hepatocytes, FD; Fat Droplets

**Fig. 9:**
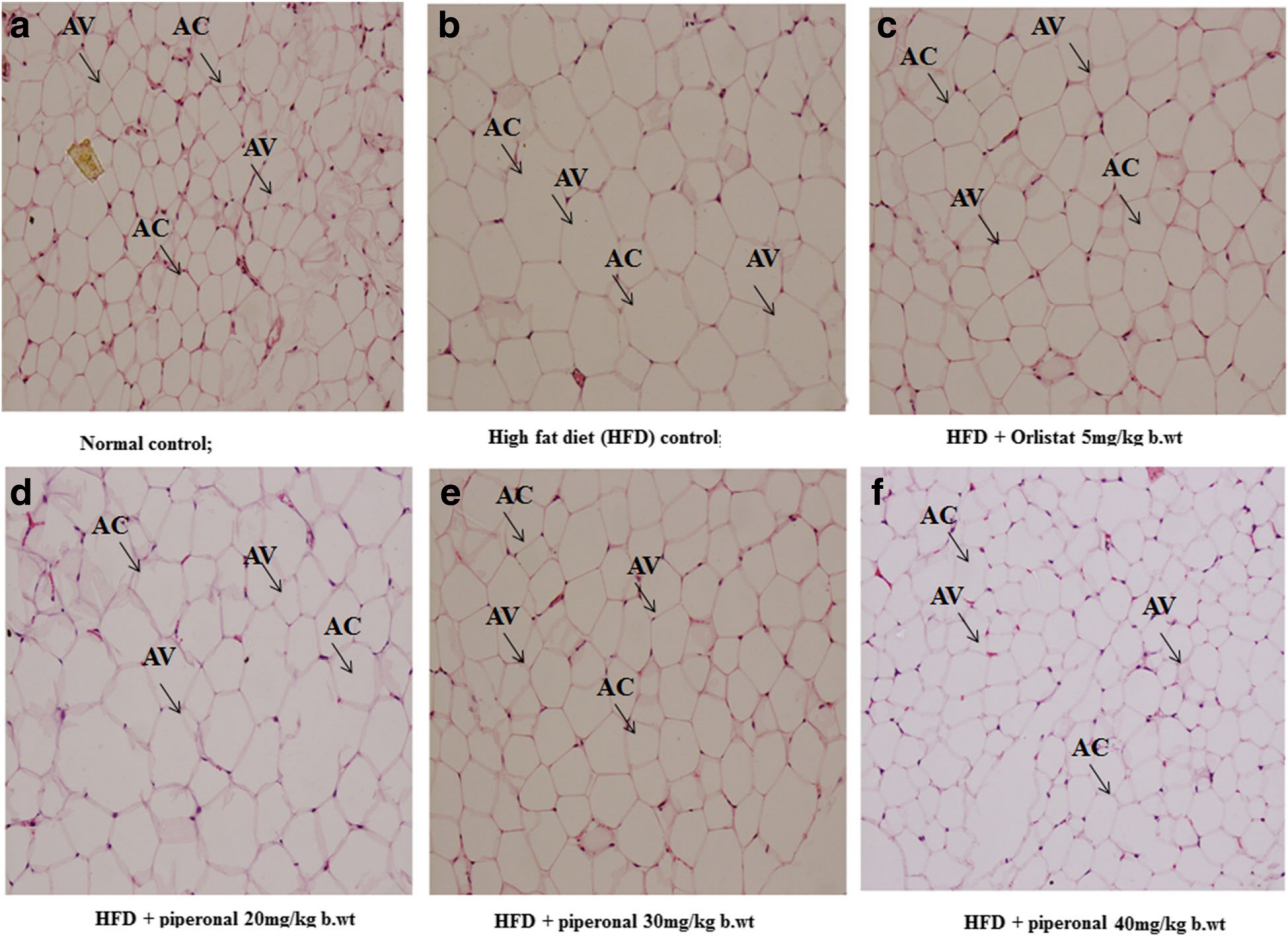
Effect of piperonal on adipose tissue histology of control and experimental obese rats. Normal control; (**a**) Adipose tissue histological observation clearly showed normal cell size. (**b**) HFD control rats showed enlarged adipocytes; Supplementation of orlistat and piperonal at different doses 20, 30, 40 mg/kg b.wt decreased fat cell size and volume (Fig. 9 **c**, **d**, **e**, **f**). AC: Adipocyte Cell; AV: Adipocyte Volume

**Fig. 10:**
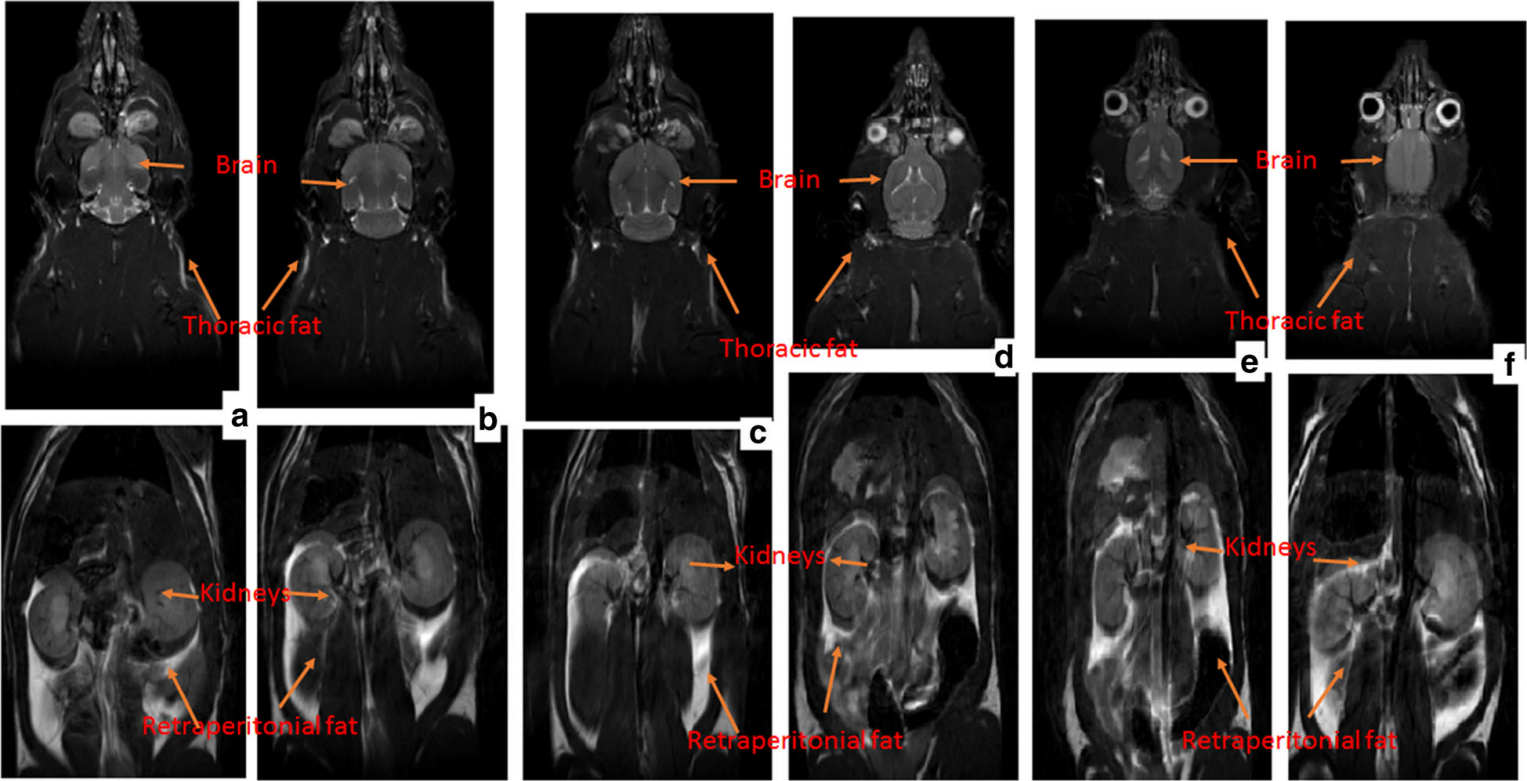
Effect of piperonal on adipose tissue distribution in control and experimental obese rats. Extensive distribution of fat in HFD fed rats is seen in (**b**) (HFD control), Supplementation of orlistat (5 mg/kg b.wt) and piperonal at different doses 20, 30, 40 mg/kg b.wt decreased fat content and distribution (**c**, **d**, **e**, **f**). **A**). Normal Control, **B**). High Fat Diet (HFD) Control, **C**). HFD+Orlistat 5mg/kg b.wt, **D**). HFD+Piperonal 20mg/kg b. wt, **E**). HFD+Piperonal 30mg/kg b. wt, **F**) HFD+Piperonal 40mg/kg b. wt

### Histopathological observations

In histological examination, the liver of HFD-fed rats showed signs of hepatic steatosis with severe swelling of hepatocytes and fat accumulation (Fig-8a and b). Interestingly, the liver sections made from piperonal (40 mg/kg b.wt)-treated rats did not show intense changes as that of HFD-fed control rats indicating its protective effect on them, though insufficient. On the same lines, the histology sections of adipose tissue of HFD-fed rats showed hypertrophy and hyperplasia of adipocytes when compared with normal control group (Fig. 9b). Supplementation of piperonal decreased fat cell size and volume in a dose-dependent manner, with the maximum therapeutic effect being observed at 40 mg/kg b.wt, as can be seen in Fig. 9c–f, indicating the antiadipogenic activity of piperonal.

### Adiposity distribution by 7 tesla magnetic resonance imaging

7 T–MRI was used to measure the magnitude of adipose tissue formed, its distribution and its effect on other organ volumes (brain and kidney). Extensive distribution of fat in HFD-fed rats is seen (Fig. 10b, HFD control); however, supplementation of piperonal decreased retroperitoneal fat content, its distribution and increased brain volume in a dose-dependent manner, with the maximum therapeutic effect being observed at 40 mg/kg b.wt (Fig. 10c–f).

## Discussion

A number of advances in our understanding of the physiological and molecular basis of appetite and body weight regulation have come from animal models. Diet-induced obesity (DIO) in animals shares many features with human obesity and hence they, more particularly rodent models, are extensively used to find efficient functional foods or their active ingredients for preventing and/or reducing obesity and associated co-morbidities [23, 37–39]. In the present study, HFD-fed group displayed significantly higher body weight gain compared to normal diet-fed group, which is the hallmark of obesity [37]. There is a good rapport between body weight and its composition in relation to organ weights and the menace of metabolic disorders [40]. In the present study, the inclusion of piperonal in diet showed a more significant reduction in body weight when compared with HFD-fed control rats. Interestingly, piperonal had no apparent suppressive effect on feeding patterns of HFD-fed rats, which indicates that piperonal-induced weight reduction involves a mechanism that is independent of the amount of food consumed by the animals. The drop in body weight may be due to several mechanisms like enhanced thermogenesis, reduced lipid digestion-transport, reduced lipogenesis-adipogenesis and increased lipolysis or energy expenditure [2].

In the body, adipose tissue is a key site of energy storage and is imperative for energy homeostasis. Nevertheless, long-term consumption of HFD causes obesity and insulin resistance possibly by reducing the interaction between insulin and insulin receptor substrate-1 (IRS-1) via diacylglycerol signaling [38, 41]. In the present study, rats fed with HFD have developed insulin resistance as indicated by amplified plasma insulin and glucose (Table 3), which is possibly due to impairment in the regulation of insulin-mediated glucose uptake in skeletal muscles or possibly through regulation of cell energy metabolism or reducing free fatty acids. Our results with piperonal demonstrating its insulin sensintizing activity are in line with the studies of Uma et al. [38] and Li et al. [25].

Leptin and adiponectin are the two most studied adipocyte-secreted hormones which have a major influence on energy balance and their measurement in plasma level may designate the compassion of an animal-to-weight gain when exposed to an HFD [15, 42]. Leptin secretion is directly proportional to adipose tissue mass as well as nutritional grade [21], whereas adiponectin is inversely correlated with body mass [17]. In the present study, feeding on HFD increased body weight and fat mass, despite an effective decrease in the circulating level of adiponectin and increase of the anorexigenic hormone leptin, a finding reliable with previous reports [43]. Interestingly, piperonal treatment decreased leptin and enhanced adiponectin secretions in plasma of HFD-induced obese rats. This may be due to downregulation of leptin and upregulation of adiponectin by piperonal at the molecular level in adipose tissue [44, 45].

One of the key strategies to suppress obesity is to inhibit the digestion and assimilation of the dietary fats and sugars [43]. Amylase and lipase, the key enzymes in carbohydrate and lipid digestion, are utilized as targets in drug design in an effort to treat obesity and associated disorders. In our study, elevated levels of amylase and lipase were recorded in HFD-fed rats, which were subsequently lowered by piperonal supplementation. So, suppression of these digestive enzymes is therefore beneficial in the treatment of obesity [46].

Antihyperlipidemic and antiobesity effects in animals and humans have become an important issue for molecular nutrition and food research. Supplementation of piperonal significantly lowered TC, TGs, FFAs and PLs in both plasma and liver and LDL-C levels in plasma of HFD-induced obese rats. An earlier report [39] has shown that supplementation of piperonal to HFD-fed animals leads to a hypolipidemic state by decreasing cholesterol absorption from the intestine which leads to lowered availability of FFAs to the liver.

Lipid homeostasis is maintained by lipogenesis and lipolysis, which are strongly associated with obesity development, fatty liver, insulin resistance and type-2 diabetes [43]. During these metabolic derangements, alteration of cell biochemistry and function is known to involve dysregulation of key factors responsible for cell signaling, such as membrane receptors, kinases, phosphatases and transcription factors [47]. The present study indicates that HFD-induced obesity in rats occurs concomitantly with major changes in transcription factors like elevated mRNA expression of PPARγ in adipose tissue and its target genes FAS, Fab-4 and activation of the proinflammatory factor TNF-α; the effects of inflammation in adipose tissue are not limited to insulin signaling alone. In addition, inflammatory signaling in the adipocyte can also upregulate the activity of PPARγ, which is responsible for adipogenesis. PPARγ is known to regulate the transcription of FAS and Fab-4. Fatty-acid synthase, a key lipogenic enzyme, catalyzes the biosynthesis of long chain fatty acids from acetyl-CoA precursors and is activated by binding to its promoter region by SREBP1. Fab-4 is an intracellular fatty acid-binding protein that binds long-chain fatty acids with high affinity. UCP-2, a fat-burning protein, acts as the main regulator of thermogenesis. Elevated plasma FFA, an exogenous ligand for PPARγ, can activate PPARγ through direct interaction with the ligand-binding domain during obesity [48]. It is well known that PPARγ is activated by SREBP1c that leads to decreased FFA oxidation, increased FAS activity and increased lipogenesis. In the present study, supplementation of piperonal altered the expression of PPARγ, FAS, Fab-4, SREBP-1c, TNF-α and UCP-2 to near-normal level in a dose-dependent manner. The reversal effects of piperonal on the expressions of FAS, SREBP-1c, Fab-4, and TNF-α indicate its antiobesogenic activity through regulation of master regulator PPARγ. Elevated expression of UCP-2 with piperonal (40 mg/kg b.wt) treatment shows the thermogenic nature of piperonal causing dissipation of energy through heat. Thus, our findings indicate that treatment of piperonal can attenuate HFD-induced alterations by working on multiple targets [39].

Obesity and hyperlipidemia synergistically endorse systemic oxidative stress disparity between tissue-free radicals, reactive oxygen species (ROS) and antioxidants. Hyperglycemia, elevated lipid levels, inadequate antioxidant defenses and hyperleptinemia could be the possible mechanisms that generate oxidative stress during obesity. ROS could react with polyunsaturated fatty acids, which leads to lipid peroxidation, a renowned parameter for assessing oxidative stress. In the present study, elevated plasma TBARS levels in HFD-induced obese rats suggested enhanced lipid peroxidation leading to tissue damage and incapability of antioxidant defense mechanisms to avert free radical attack. Intracellular antioxidant enzymes and TBARS are the specific markers of oxidative damage at the cellular level [26, 47]. An earlier report by us and others depicted that the lowered level of cellular oxidative damage is connected with multiple enzymatic (SOD, CAT, GPx) and non-enzymatic antioxidant (GSH) defense systems present in cells [18, 26, 43]. Unremitting treatment with Piperonal considerably improved the levels of endogenous antioxidant enzymes (SOD, CAT, GPx) and non-antioxidant enzyme (GSH), and prohibited membrane damage by diminishing lipid peroxidation compared to HFD control. This was further supported by histopathological analysis of liver and adipose tissues.

In the present study, histopathological examination of the liver of the HFD rats showed microvesicular steatosis in hepatocytes as well as more amounts of fat droplets. Piperonal treatment at 40 mg/kgb.wt ameliorated these hepatic lesions partially. Similarly, the prominent changes observed in adipocyte sizes and volume of HFD-fed rats are considerably alleviated in piperonal and Orlistat-treated groups.

## Conclusions

The present study substantiates that piperonal effectively inhibited adipogenesis possibly by acting on transcription factors like PPARγ and its target genes like FAS, UCP2 and Fab-4, and pro-inflammatory factors in adipose tissues. These modes of action on modulating genes associated with adipogenesis and lipid metabolism by piperonal are considered relatively novel when compared with the mechanisms reported hitherto with other anti-obesity phytochemicals. Finally, we conclude that piperonal attenuates obesity and associated ailments by working on multiple targets and paves the way to develop a novel herbal formulation.

## Abbreviations

ACC: Acetyl-CoA carboxylase
BMC: Bone mineral concentration
BMD: Bone mineral density
DXA: Dual X ray absorptiometry
Fab-4: Fatty acid binding protein-4
Fas: Fatty acid synthase
HDL: High density lipoproteins
HFD: High fat diet
HMG-CoA reductase: 3-hydroxy-3-methyl-glutaryl-coenzyme A reductase
HPLC: High-performance liquid chromatography
LDL: Low density lipoproteins
NMR: Nuclear magnetic resonance spectroscopy
PPAR-ϒ: Peroxisome proliferator-activated receptor gamma
SD rats: Sprague Dawley rats
SREBP-1c: Sterol regulatory element-binding transcription factor 1c
TC: Total cholesterol
TG: Total triglycerides
TNF-α: Tumor necrosis factor-α
TOBEC: Total body electrical conductivity
UCP: Uncoupling protein-2
VLDL: Very low density lipoproteins
