# Correction to: Antiobesity potential of Piperonal: promising modulation of body composition, lipid profiles and obesogenic marker expression in HFD-induced obese rats

**DOI:** 10.1186/s12986-017-0232-0

**Published:** 2017-12-06

**Authors:** Balaji Meriga, Brahmanaidu Parim, Venkata Rao Chunduri, Ramavat Ravindar Naik, Harishankar Nemani, Pothani Suresh, Saravanan Ganapathy, V. V. Sathibabu Uddandrao

**Affiliations:** 1 Animal Physiology and Biochemistry Laboratory, Department of Biochemistry, Sri Venkateswara University, Tirupati, Andhra Pradesh 517502 India; 2 Present Address: Department of Bio-Technology, VSU College of Sciences, Vikrama Simhapuri University, Nellore, Andhra Pradesh 524320 India; 3 Department of Chemistry, Sri Venkateswara University, Tirupati, Andhra Pradesh 517502 India; 4 National Center for Laboratory Animal Sciences, National Institute of Nutrition (Indian Council of Medical Research), Hyderabad, India; 5 Department of Biochemistry, Center for Biological Sciences, K. S. Rangasamy College of Arts Science, Tiruchengode, Tamil Nadu India

## Correction

Following publication of the original article [1], the authors requested a correction to the name of one of the co-authors. The correct name and spelling is V.V. Sathibabu Uddandrao.

The original article has been updated.
